
JOURNAL INFORMATION
==============================
NLM Title Abbreviation: ScientificWorldJournal
Journal ID: ScientificWorldJournal
NLM Journal ID: 101131163
Journal ID: TSWJ

The Scientific World Journal

ISSN: 2356-6140
EISSN: 1537-744X
Publisher: Wiley

ARTICLE INFORMATION
==============================
PMCID: PMC5936537
PMID: 29861681
DOI: 10.1100/tsw.2005.72
Article ID: 465284
Article version: 1
Subjects: Editorial

Biochemistry and Molecular Biology-Aims and Scope

Learmonth Robert P.
University of Southern Queensland, QLD, Australia
Publication date: 2005
Electronic publication date: 2005 Jul 5
Volume: 5
First page: 500
Last page: 500
Copyright: Copyright © 2005 Robert P. Learmonth.
Copyright year: 2005
License: This is an open access article distributed under the Creative Commons Attribution License, which permits unrestricted use, distribution, and reproduction in any medium, provided the original work is properly cited.
License URL: https://creativecommons.org/licenses/by/3.0/

==============================
Editorial
*TheScientificWorldJOURNAL* (2005) 5, 500
ISSN 1537-744X; DOI 10.1100/tsw.2005.72

### Biochemistry and Molecular Biology - Aims and Scope

Robert P. Learmonth
*University of Southern Queensland, QLD, Australia*

E-mail: learmont@usq.edu.au

*Published July 5, 2005*

---

TSW Biochemistry and Molecular Biology welcomes submissions on all aspects of biochemistry and molecular biology, including original research papers, reviews and mini-reviews, short communications, methods and protocols, editorials and commentaries. The scope of this journal is wide ranging, some examples of topics might include general biochemistry, physical biochemistry, structure and dynamics of proteins, nucleic acids, carbohydrate or membranes, gene structure and expression, enzymology, bioenergetics, metabolic biochemistry, genomics and proteomics. This list is not exclusive, and authors are encouraged to submit articles on any aspect of biochemistry and molecular biology of micro organisms, plants, animals or humans. We encourage cross-linking articles with other relevant domains. In line with TheScientificWorldJOURNAL general policy, this domain seeks to enable rapid and wide dissemination of peer-reviewed publications.

.

---

**This article should be referenced as follows:**

Learmonth, Robert P. (2005) Biochemistry and Molecular Biology – Aims and Scope. *TheScientificWorldJOURNAL* **5**, 500.

---

©2005 with author.
